# The universal characteristics of a thermodynamic model to conform to the Gibbs-Duhem equation

**DOI:** 10.1038/srep35792

**Published:** 2016-10-20

**Authors:** Dong-Ping Tao

**Affiliations:** 1 Faculty of Metallurgical and Energy Engineering, Kunming University of Science and Technology, Kunming 650093, Yunnan, P.R. China

## Abstract

In a multi-component homogeneous system, the relationship between partial molar and molar quantity (RPMQ) is proved to be an equivalent relation of the Gibbs-Duhem equation. The universal characteristics of a thermodynamic model to conform to the Gibbs-Duhem equation are inferred from the RPMQ. Based on the inference, an asymmetric regular solution model is suggested to deal with those systems that exhibit strong negative deviation, strong positive deviation, and both strong positive and negative deviation from ideality.

In the fields of solution thermodynamics and multiphase equilibria, a universal thermodynamic model still has not been found so far that can be applied to all the gas, liquid or solid mixtures. Some researchers are often faced with the existing thermodynamic models not to be suitable for an interested practical system. So they have to construct a better model by using classical and statistical thermodynamics, molecular physics and mathematics for solving some pressing thermodynamic and kinetic problems in the system, such as prediction of unknown data of thermodynamic properties, determination of various compositions at a multiphase equilibrium, estimation of chemical potentials of diffusion processes between different phases and so forth.

However, the constructed physical and mathematical models must be verified by using the Gibbs-Duhem equation. Otherwise they are thermodynamically incorrect. This is one of the main obstacles of constructing a thermodynamic consistent model. The model researcher should have a failed experience that an appearing rational elaborated model was finally abandoned because of its absence of thermodynamic consistency. It results in a question: how efficiently does the researcher construct a thermodynamic model to conform to the Gibbs-Duhem equation? That is to say: what universal characteristics should a thermodynamic consistent model have? Unfortunately, this question has not been solved yet up to now. In fact, the universal characteristics are not found in literatures.

In this paper, the author tries to prove an equivalent relation of the Gibbs-Duhem equation and infer the universal characteristics from it, and then based on the inference to construct an unreported interested thermodynamic model for solving the fitting problems of component activity curves in the systems to be of strong deviations from ideality.

## Results

For a multi-component homogeneous system, once a molar fraction of arbitrary component *t*, 
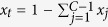
, is chosen as a subordinate variable, the relationship between partial molar and molar quantity (RPMQ) at constant temperature and pressure are generally formalized as


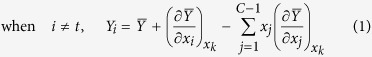



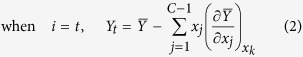


or both of them are combined as^1^


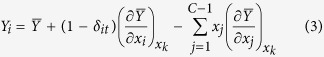


where *δ*_*it*_ is the Kronecker delta (*δ*_*it*_ = 0 if *i* ≠ *t*, and *δ*_*it*_ = 1 if *i* = *t*).

The RPMQ is proved to be an equivalent relation of the Gibbs-Duhem equation at constant temperature and pressure


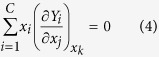


where *C* is the number of components, 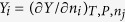 is the partial molar quantity of component *i*, *Y* is an extensive property of the system, 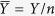 is the molar quantity of the extensive property for one mole of the system, 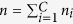 is the total number of moles of the system, *n*_*i*_ is the number of moles of component *i*, and *x*_*i*_ = *n*_*i*_/*n* is the molar fraction of component *i*. Here note that the subscripts of the temperature *T* and pressure *P* of all partial derivatives may be omitted for concisely writing.

From the RPMQ, the universal characteristics of a thermodynamic model to conform to the Gibbs-Duhem equation are inferred as follows:

- it is not divergent or has no problem of infinite in the entire compositions.
- its first order partial derivative exists.
- when a molar fraction of arbitrary component approaches one unit, it can revert to the molar quantity of the pure component.

In other words, based on the mathematical and physical characteristics of the RPMQ and molar quantity, an inference states that for a multi-component homogeneous system, only if any thermodynamic model of the molar quantity is not divergent or has no problem of infinite in the entire compositions, and its first order partial derivative exists, and it satisfies the pure-component boundary condition (*x*_*i*_ = 1 or all other *x*_*j*≠*i*_ = 0), the model conforms to the Gibbs-Duhem equation.

## Discussion

The RPMQ is an important relation in the fields of solution thermodynamics and multiphase equilibria. It has been widely used to derive all partial molar quantities from a molar quantity expressed by various thermodynamic models in a multi-component homogeneous system, such as electrolyte solutions, polymer or metallurgical melts, rock-magma, solid solutions, gas mixtures, and so on. Although it had different forms, they were demonstrated to be equivalent to each other^2^. However, few people know that the RPMQ is equivalent with the Gibbs-Duhem equation so far. About a decade ago, a conjecture was proposed that for the molecular entity vacancy model (MEVM) its partial molar excess Gibbs energies derived from the RPMQ suffice the Gibbs-Duhem equation^3^. But its proof still has not been reported up to now.

Here, it can be proved that the RPMQ is an equivalent relation of the Gibbs-Duhem equation at constant temperature and pressure as follows:

For an extensive property *Y* of the system, its molar quantity 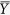 can be written as


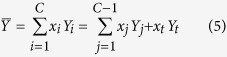


where 
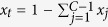
 is chosen to be a subordinate variable which needs special attention, namely, the number of independent variables of 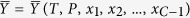 is the *C* − 1 and diminishes one in comparison with the number of independent variables of *Y* = *Y*(*T*, *P*, *n*_1_, *n*_2_, ..., *n*_*C*_). This is a key difference between 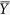 and 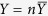.

Substituting equation (5) into equation (1), one can get


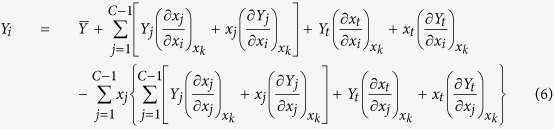


Here note that in the first sum term of right side of equation (6) the number of variable *x*_*i*_ is equal to that of variable *x*_*j*_ due to the condition of *i* ≠ *t* in equation (1), i.e., both of them are the *C* − 1; similarly, in the second sum term of right side of equation (6) the number of variable *x*_*j*_ of the sum symbol outside the braces is equal to that of variable *x*_*j*_ of the sum symbol inside the braces, i.e., both of them also are the *C* − 1. That is to say that the number of subscript *i* is equal to that of subscript *j* in equation (6).

Thus, considering 
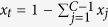
, there exist three cases of the partial derivatives of *x*_*j*_ and *x*_*t*_ with respect to *x*_*i*_ in the first row of equation (6) respectively:


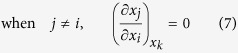



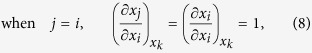



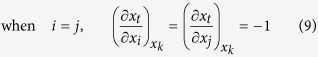


Similarly, considering 
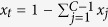
, there also exist three cases of the partial derivatives of *x*_*j*_ and *x*_*t*_ with respect to *x*_*j*_ in the second row of equation (6) respectively:


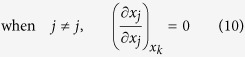



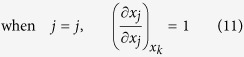



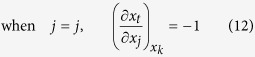


Then, substituting equations (5), (7) to (12) into equation (6), one can get


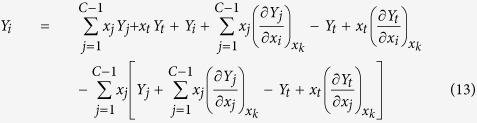


Combining similar terms in equation (13), that is


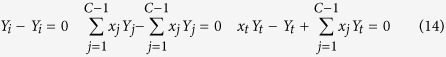



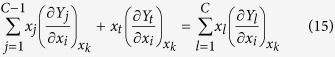



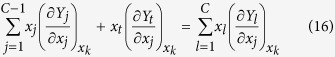


Then, equation (13) becomes


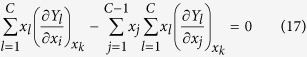


Here note that the subscript *i* is completely same as the subscript *j* in equation (17) due to the condition of *i* ≠ *t* in equation (1). Thus, from equation (17) one can get


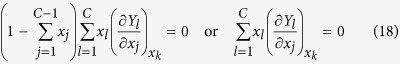


Obviously, this result exactly is the Gibbs-Duhem equation or the equation (4) because the number of subscript *l* in equation (18) is completely same as that of subscript *i* in equation (4).

Similarly, substituting equation (5) into equation (2), one can get


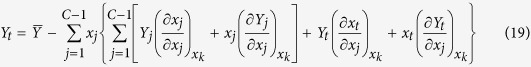


Here note that in the sum term of right side of equation (19) the number of variable *x*_*j*_ of the sum symbol outside the braces is equal to that of variable *x*_*j*_ of the sum symbol inside the braces, i.e., both of them are the *C* − 1.

Similarly, considering 
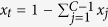
, there also exist three cases of the partial derivatives of *x*_*j*_ and *x*_*t*_ with respect to *x*_*j*_ in the second term of equation (19) respectively:


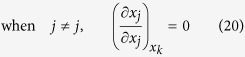



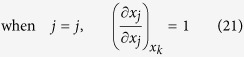



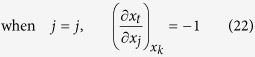


Then, substituting equations (5), (20) to (22) into equation (19) and combining similar terms, one can get


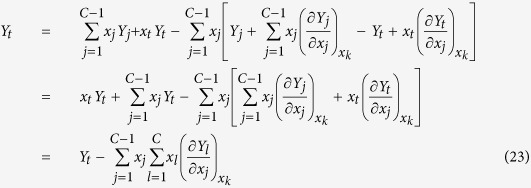


where the similar terms combined are


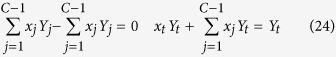



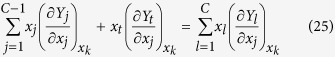


Thus, from equation (23) one can get


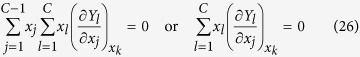


Obviously, this result also is the Gibbs-Duhem equation or the equation (4) because the number of subscript *l* in equation (26) is completely same as that of subscript *i* in equation (4).

From the proof, it can be seen that the equations (1) to (3) are an equivalent relation of equation (4) indeed. That is to say that the RPMQ has guaranteed thermodynamic consistency of the partial molar quantities. Consequently, the molar quantity expressed by a thermodynamic model certainly conforms to the Gibbs-Duhem equation if it satisfies the PRMQ.

Based on the mathematical and physical characteristics of the RPMQ and molar quantity, therefore, an inference states that for a multi-component homogeneous system, only if any thermodynamic model of the molar quantity is not divergent or has no problem of infinite in the entire compositions, and its first order partial derivative exists, and it satisfies the pure-component boundary condition (*x*_*i*_ = 1 or all other *x*_*j*≠*i*_ = 0), the model conforms to the Gibbs-Duhem equation. The given mathematical and physical restraints are able to guide the researcher to construct a thermodynamic consistent model which depends on composition at constant temperature and pressure.

For the constructed thermodynamic model, what the researcher needs to do next is only to check whether its partial molar quantities derived from the RPMQ have errors. The checking method is to substitute them into the sum formula or equation (5). If they are consistent with their molar quantity expressed by the model, they are correct; otherwise, they are incorrect. It can be seen that because equation (4) contains the second order partial derivatives of the molar quantity with respect to the molar fractions, the checking method of using equation (5) is much simpler and easier than that of using equation (4).

## Methods

In order to verify the above inference, and meanwhile, in order to search for a thermodynamic model that is suitable for those systems exhibiting strong negative deviation, strong positive deviation, and both strong positive and negative deviation from ideality, the author tries to construct this kind of model according to the universal characteristics as follows:

Based on the regular solution model (RSM)^1^, 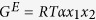, where *G*^*E*^ is the molar excess Gibbs energy of the 1–2 binary system, *x*_1_ and *x*_2_ are the molar fractions of components 1 and 2 respectively, *α* is the RSM parameter, *T* is the absolute temperature and *R* is the gas constant. Since the RSM is a symmetric model at vertical line *x*_2_ = 0.5, an asymmetric regular solution model (ARSM) can be empirically constructed by expressing the parameter *α* as a function of compositions:


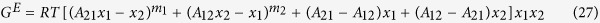


Obviously, the RSM parameter *α* becomes


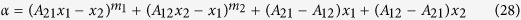


where *m*_1_, *m*_2_, *A*_21_ and *A*_12_ are the ARSM parameters. Here note that the value ranges of *m*_1_ and *m*_2_ are the improper fraction to be larger than one unit or positive integer, and the value ranges of *A*_21_ and *A*_12_ are real number. Substituting equation (27) into equations (1) and (2) or equation (3), the partial molar excess Gibbs energies 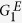 and 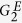 can be respectively derived as


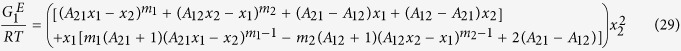



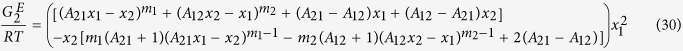


It is easily checked that equations (27), (29) and (30) are not divergent or have no problem of infinite in whole composition of the 1–2 binary system, and they satisfy the pure-component boundary condition: when *x*_1_ = 1 or *x*_2_ = 1, then 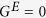, 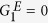 and 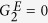. Substituting equations (29) and (30) into the sum formula of the molar excess Gibbs energy: 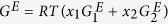, both of them are checked to be consistent with equation (27), namely, they are correct. This shows that the ARSM conforms to the Gibbs-Duhem equation.

The next work is to examine whether the ARSM is suitable for those strong deviation binary systems. Let us select some representative liquid alloys, such as the Al-Au at 1400 K, Li-Na at 600 K and B-Nd at 3000 K^4^, and compare the ARSM and the molecular interaction volume model (MIVM)^5^ with literature data of component activities in the binary alloys.

For the MIVM in the 1–2 binary system, its simplified forms^6^ of 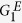 and 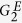 can be expressed as respectively


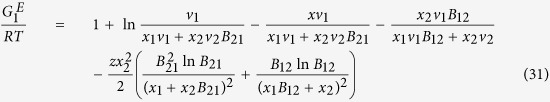



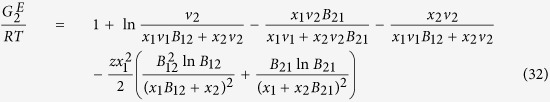


where *z* = 10 is the coordination number of component *i*, *v*_*i*_(cm^3^/mol) is the molar volume of component *i* at solid state, *B*_21_ and *B*_12_ are the MIVM parameters which can be determined by fitting binary activity data. For the components Al, Au, B, Li, Na and Nd in the above three alloys, their molar volumes are 10.00, 10.21, 4.394, 12.97, 23.71 and 20.59 cm^3^/mol, respectively.

**Figure 1:**
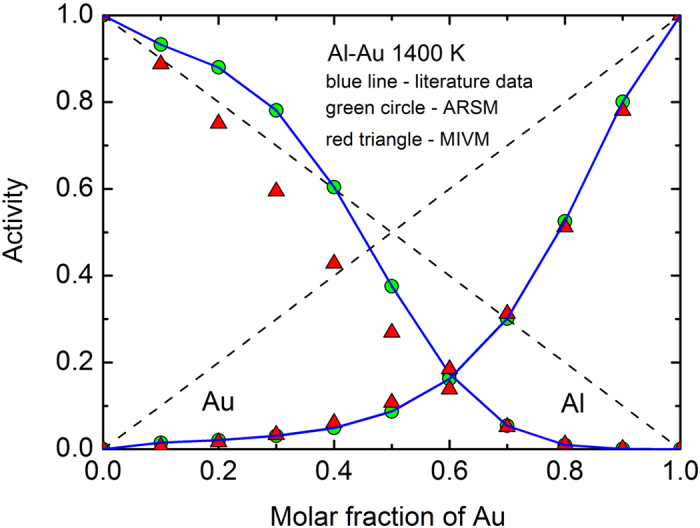
Comparison of fitting errors of component activities with ARSM and MIVM in the Al-Au liquid alloys at 1400 K: blue line – literature data^4^, green circle – ARSM, red triangle – MIVM, and dash line – ideal line: *a*_*i*_ = *x*_*i*_.

**Figure 2:**
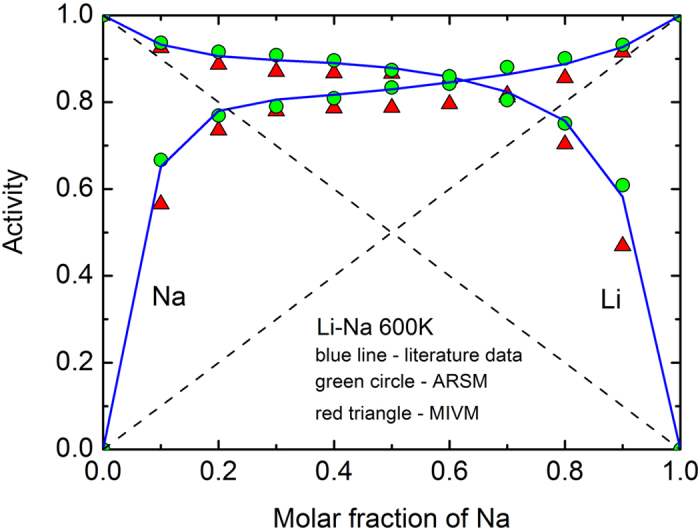
Comparison of fitting errors of component activities with ARSM and MIVM in the Li-Na liquid alloys at 600 K: blue line – literature data^4^, green circle – ARSM, red triangle – MIVM, and dash line – ideal line: *a*_*i*_ = *x*_*i*_.

**Figure 3:**
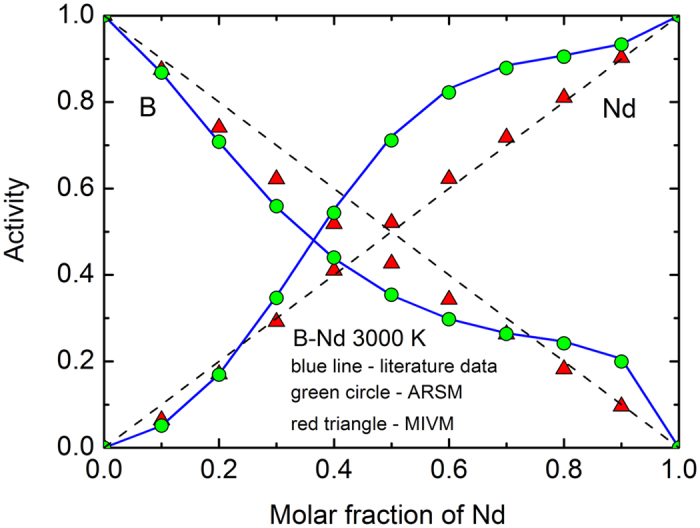
Comparison of fitting errors of component activities with ARSM and MIVM in the B-Nd liquid alloys at 3000 K: blue line – literature data^4^, green circle – ARSM, red triangle – MIVM, and dash line – ideal line: *a*_*i*_ = *x*_*i*_.

The fitting results are shown in Table 1 and Figs 1, 2 and 3 where the average relative error is 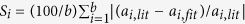, where 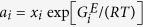 is the activity of component *i*, *a*_*i*,*lit*_ and *a*_*i*,*fit*_ are the literature data and the fitted values of activity of component *i* respectively, and *b* is the number of data.

It can be seen that when *m*_1_ = *m*_2_ = 1, equations (27), (29) and (30) are suitable for the Al-Au at 1400 K which exhibits strong negative deviation including a part positive deviation of the Al activity curve from ideality, and they are suitable for the Li-Na at 600 K which exhibits strong positive deviation from ideality; when *m*_1_ = 1 and *m*_2_ = 2, they are suitable for the B-Nd at 3000 K which exhibits both positive and negative deviations from ideality. All the fitting errors of component activities of the ARSM are much smaller than that of the MIVM, especially for the Al-Au at 1400 K and the B-Nd at 3000 K. The results indicate that the ARSM has ability to deal with quite asymmetric activity curves, whereas the MIVM appears to be not suitable for the cases. So it is necessary to modify the MIVM for improving its fitting accuracy further.

The example shows that in order to solve some thermodynamic problems in a practical system, if a researcher is able to follow the premise of the universal characteristics above and tries to construct various forms of an unreported model based on the knowledge of physics and mathematics, a valuable thermodynamic consistent model could be finally accomplished. This is not as difficult as one usually thinks.

## Additional Information

**How to cite this article**: Tao, D.-P. The universal characteristics of a thermodynamic model to conform to the Gibbs-Duhem equation. *Sci. Rep.* **6**, 35792; doi: 10.1038/srep35792 (2016).

## Figures and Tables

**Table 1:** Parameters and fitting errors of ARSM and MIVM in the binary liquid alloys.

Alloy	*T*(K)	*A*_21_	*A*_12_	*B*_21_	*B*_12_	*S*_1_(%)		*S*_2_(%)	
						ARSM	MIVM	ARSM	MIVM
*m*_1_ = 1	*m*_2_ = 1								
Al-Au	1400	−2.11	−4.06	0.43	2.47	1.63	16.6	0.58	16.7
B-Nd	3000	0.55	1.48	0.96	0.89	0.79	18.1	0.86	18.5
*m*_1_ = 1	*m*_2_ = 2								
Li-Na	600	−0.33	−2.35	0.87	0.76	1.52	5.08	1.43	6.03
